# Safety and Prognostic Impacts of Ovarian Preservation during Radical Hysterectomy for Early-Stage Adenocarcinoma and Adenosquamous Cervical Cancer

**DOI:** 10.1155/2020/5791381

**Published:** 2020-11-12

**Authors:** Arisa Theplib, Jitti Hanprasertpong, Kittinun Leetanaporn

**Affiliations:** ^1^Department of Obstetrics and Gynecology, Faculty of Medicine, Prince of Songkla University, Songkhla 90110, Thailand; ^2^Department of Biomedical Sciences, Faculty of Medicine, Prince of Songkla University, Songkhla 90110, Thailand

## Abstract

**Objective:**

To identify the incidence of ovarian metastasis and the impact of ovarian preservation on oncological outcomes for early-stage adenocarcinoma and adenosquamous cervical cancer.

**Methods:**

281 patients with stages IA2-IB1 adenocarcinoma and adenosquamous cervical cancer who underwent radical hysterectomy with pelvic lymphadenectomy (RHND) were included in the study. The incidence of ovarian metastasis was evaluated from 173 patients who underwent oophorectomy during RHND. Subgroup analysis was performed for patients less than 50 years (196 of 281 patients) who were classified into two groups, ovarian preservation and nonovarian preservation groups. 5-year recurrence-free survival (5-yr RFS) and 5-year overall survival (5-yr OS) were evaluated and compared between these groups.

**Results:**

There was no evidence of ovarian metastasis, synchronous ovarian cancer, or ovarian recurrence during follow-up. In patients less than 50 years of age, there were no statistically significant differences in the 5-yr RFS (*P* = 0.363), or 5-yr OS (*P* = 0.974) between the ovarian preservation and nonovarian preservation groups. In Kaplan–Meier analysis, the ovarian preservation group seemed to have a slightly better OS in long-term follow-up (after 15 years); however, the difference was not statistically significant.

**Conclusions:**

Ovarian preservation was safe in adenocarcinoma and adenosquamous cervical cancer stages IA2-B1. However, the impact of ovarian preservation on oncological outcomes needs to be further investigated.

## 1. Introduction

Overall, the peak incidence of age at the diagnosis of cervical cancer is during the premenopausal period. However, in recent years, these cancers have been occurring in earlier stages of life than previously [1, 2]. Also, although the overall incidence of cervical cancer has declined since cervical cancer screening programs and human papillomavirus vaccine has become available, the proportion of adenocarcinoma relative to squamous cell carcinoma and all cervical cancers has been increasing [1–4]. Compared with squamous cell carcinoma, patients with adenocarcinoma tend to be younger [4, 5] and associated with an equal or poorer prognosis [6–9]. Patients with adenocarcinoma seem to exhibit greater hematogenous spread. This apparent rise of incidence coupled with the poor prognosis of adenocarcinoma is of importance for treatment. Although the current treatment algorithm is the same as for squamous cell carcinoma, some studies have suggested that modified therapeutic strategies for adenocarcinoma need to be developed [7, 8]. The standard surgical treatment of early-stage cervical cancer is radical hysterectomy with pelvic lymphadenectomy (RHND) [10].

In younger aged patients, ovarian preservation (OP) during the RHND is aimed at preserving the hormonal function, which has an effect on women's menopausal symptoms or quality of life, osteoporosis, and cardiovascular disease [11–26]. Considering the cardiovascular protection of the ovarian hormonal effects, OP may decrease mortality from cardiovascular diseases [12–15]. However, OP in cervical cancer is not part of the routine practice, due to the risk of ovarian metastasis [17–20, 22, 23, 25]. The effects on oncological outcome are also still not clear [16, 23–26].

Several previous studies reported that the incidence of ovarian metastasis in patients with early-stage cervical cancer ranged from 0.3 to 0.7% in squamous cell carcinoma [16, 17, 19] and as high as 1.7-4.4% in adenocarcinoma [16–20]. These studies concluded that ovarian metastasis occurred in adenocarcinoma and adenosquamous carcinomas more than in squamous cell carcinomas. OP can be safe in squamous cell carcinomas, but in adenocarcinoma and adenosquamous carcinomas, it is still controversial [16–23]. Gynecologic oncologists also have to consider synchronous ovarian cancer, although the incidence of synchronous ovarian cancer and cervical cancer is very low, accounting for only 0.025% of all female genital cancers [27].

The effects of OP on oncological outcomes are the other important issue. In 2014, the Surveillance, Epidemiology, and End Results (SEER) database (1988-2007) study in patients with stages I-II adenocarcinoma and adenosquamous cervical cancer showed that there were no significant differences in cancer-specific survival (CSS) or overall survival (OS) between patients with bilateral salpingoophorectomy (SO) and OP [24]. These results are consistent with data obtained in a retrospective study and meta-analysis in 2016, in that OP had no effect on prognosis [25], while another recent population-based study from the SEER database (1988-2013) reported that among young patients with T1N0M0 cervical adenocarcinoma, OP was associated with better oncological outcomes [26].

The objectives of our study were therefore primarily to identify the incidence along with risk factors of ovarian metastasis in patients with early-stage adenocarcinoma and adenosquamous cervical cancer (all age groups) and secondly to determine the impact of OP on oncological outcomes, especially in patients aged less than 50 years.

## 2. Materials and Methods

**Figure 1:**
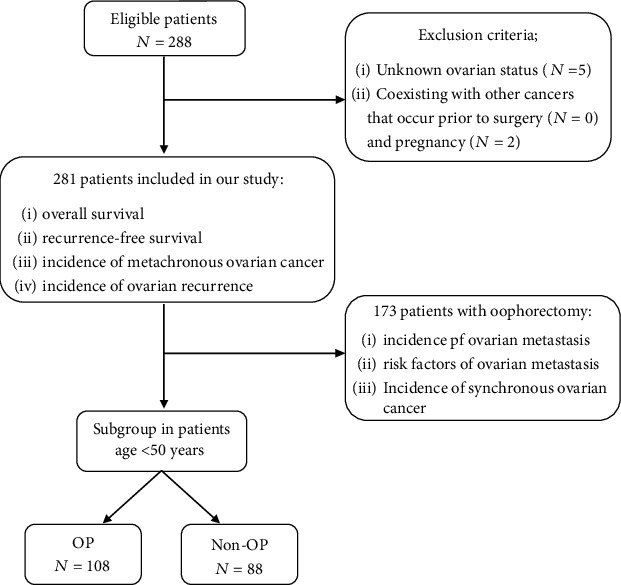
Patient inclusion and exclusion algorithm.

This study was approved by the Institutional Ethic Committee of the Faculty of Medicine, Prince of Songkla University. A retrospective medical records review was performed including all patients (*N* = 288) with stages IA2-IB1 (based on the International Federation of Gynecology and Obstetrics (FIGO) stage 2009) adenocarcinoma or adenosquamous cervical cancer who underwent a RHND at Songklanagarind Hospital between January 1987 and June 2017. Patients with unavailable data including ovarian status (*N* = 5), coexistence with other cancers that have been diagnosed prior to the surgery (*N* = 0), and pregnancy (*N* = 2) were excluded. Of all 288 patients, 281 patients met the inclusion criteria (Figure 1). Both the clinical and pathological data were obtained from the medical records, including age, FIGO stage, cervical and ovarian histology, lymphovascular invasion (LVSI), parametrial invasion, LN metastasis, vaginal margin involvement, deep stromal invasion (DSI), type of surgery, adjuvant therapy, postoperative hormonal replacement therapy (HRT), recurrence, follow-up time, and ovarian status. The incidence of ovarian metastasis was evaluated through ovarian histologic results from 173 patients who underwent oophorectomy. Synchronous ovarian cancer was also recorded in the pathology of these patients. 5-year recurrence-free survival (5-yr RFS) and 5-year overall survival (5-yr OS) were evaluated for all 281 patients.

The study included younger aged patients, who may have benefited from the preservation of ovarian hormones, and both 5-yr RFS and 5-yr OS were analyzed and compared in subgroups of patients aged less than 50 years (196 patients). As a previous study has shown that the median age of natural menopause is 51.3 years [28], patients were then classified into two groups: an OP group who did not undergo an oophorectomy and a non-OP group who underwent an oophorectomy.

Ovarian metastasis was defined as the morphology of tumor cells in ovarian tissue being the same as the cervical tumor histology obtained from the pathologic results. Synchronous ovarian cancer was defined as histopathology confirmed by 2 primary cancers of the ovaries and cervix identified at the same time. Histology of metachronous ovarian cancer was defined as the same as synchronous ovarian cancer, but occurring at a different time than the cervical cancer.

After RHND, patients in the intermediate-risk or high-risk groups were recommended to undergo postoperative adjuvant radiation (with or without concurrent chemotherapy) according to the standard criteria [9]. After completion of treatment (with or without adjuvant treatment), all patients were followed up every 3 months in the first year, every 4 months in the second year, every 6 months in the third to fifth years, and then yearly thereafter [9]. RFS was defined as the duration from the date of operation to the date of recurrence. OS was defined as the duration from the date of operation to the date of death from any cause.

The comparisons of frequency distributions between characteristic variables were analyzed using Fisher's exact, Chi-square, or Wilcoxon rank-sum test. Both survival outcomes were analyzed using the Kaplan–Meier method, and differences were compared with the log-rank test. Variables showing some evidence of association with time to the event (*P* value < 0.2) were included in initial Cox proportional hazard models, which were then refined by sequential removal of variables not contributing significantly to the scope of the model (i.e., variables having a likelihood ratio *P* value less than 0.05). All analyses were performed using the R program version 3.4.2 (R Foundation of Statistical Computing, Vienna, Austria).

## 3. Results

Of all 281 patients, the median age was 45 years, 87.2% were in stage IB1, 12.8% were in stage IA2, and most cases had adenocarcinoma histology (85.1%). The median follow-up time of all 281 patients was 5.5 years. The 5-yr RFS rate was 87.8% (83.5-92.4%), and the 5-yr OS rate was 96.3% (93.6-99.1%). Recurrence occurred in 12.5% of the patients, of which 65.7% were locoregional recurrence. However, there was no evidence of ovarian recurrence or metachronous ovarian cancer after the long-term follow-up period. In the 173 patients who underwent oophorectomy, there were no incidences of ovarian metastases or synchronous ovarian cancer in their pathological findings.

196 patients under 50 years of age, were enrolled in the subgroup analysis. The median follow-up time in this subgroup was 6.1 years. These patients were divided into 2 groups: an OP group with 108 and a non-OP group with 88 patients. There were no significant differences in demographic or clinical characteristics between these two groups, with the exception of the median age and year of treatment (Table 1). The median age was younger in the OP group than in the non-OP group (38 years vs. 45 years; *P* < 0.001). We also found that before the year 2000 the percentage of preservation of ovaries was lower than the period after (*P* = 0.008).

**Figure 2:**
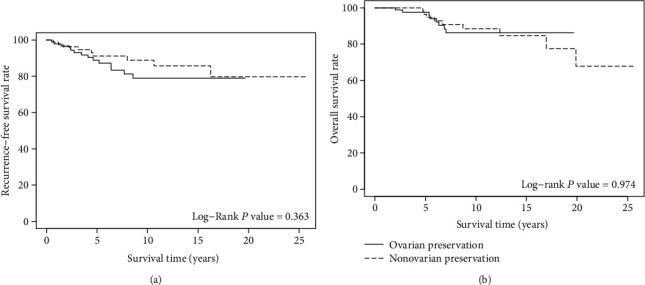
(a) Recurrence-free survival in early-stage adenocarcinoma and adenosquamous patients aged less than 50 years with ovarian preservation and nonovarian preservation. (b) Overall survival in early-stage adenocarcinoma and adenosquamous patients aged less than 50 years with ovarian preservation and nonovarian preservation.

In the Kaplan–Meier analysis, the 5-yr RFS rates of the OP and non-OP groups were 88.9% and 91.1% (*P* = 0.363) (Figure 2), and the 5-yr OS rates were 97.5% and 96.5% (*P* = 0.974) (Figure 2(b)), respectively. These differences were not significant. In univariate analysis, FIGO stage, LVSI, parametrial invasion, LN metastasis, vaginal margin involvement, DSI, and adjuvant treatment were associated with 5-yr RFS, while FIGO stage, LVSI, parametrial invasion, LN metastasis, DSI, and adjuvant treatment were significant factors for 5-yr OS (Table 2). In multivariate analysis, FIGO stage IB1 (*P* = 0.005 and *P* = 0.014 for RFS and OS, respectively) and LN metastasis (*P* < 0.001 for RFS and OS) were the significant poor prognosis factors for both 5-yr RFS (Table 3) and 5-yr OS (Table 4).

## 4. Discussion

The conservation of the ovaries for maintaining hormonal function is important in younger aged cervical cancer patients. However, OP in younger patients with early-stage adenocarcinoma and adenosquamous cervical cancer [6–9], the risk of ovarian metastasis and its effects on oncological outcomes of adenocarcinoma are still of concern. Our study found no ovarian metastasis or evidence of synchronous ovarian cancer in either early-stage adenocarcinoma or adenosquamous cervical cancer, and we therefore could not evaluate or analyze the risk factors of ovarian metastasis in this study. However, when comparing our study with previous studies, the ovarian metastasis in early-stage adenocarcinoma and adenosquamous cervical cancer ranged from 1.7 to 4.4% [16–20]. Shimada et al. reported ovarian metastasis in 546 patients and another study by Landoni et al. also reported this in 380 patients with early-stage adenocarcinoma or adenosquamous cervical cancer [17, 19]. These studies were conducted with larger sample sizes; hence, the incidence of ovarian metastasis may be considered low when compared to the ratio of the sample size in our study. This may be the reason that we could not find evidence of ovarian metastasis, which has also been noted in previous studies reporting the evidence of synchronous ovarian cancer as only 0.025% in the population database [27]. Furthermore, most of the patients in our study were younger than 50 years of age and were stage IB1 < 2 cm, which seem to be the factors that are associated with a low risk for ovarian metastasis, as noted in previous studies [17, 19]. Many clinicopathologic studies have described a number of risk factors for ovarian metastasis in cervical cancer, including older patients [17, 22], advanced FIGO stage [17, 22], LN metastasis [20–22], DSI [17, 18, 22], LVSI [21, 22], uterine invasion [20–22], parametrial invasion [17, 20, 22], tumorsize > 4cm [22, 24], and histology of adenocarcinoma [17, 20, 21]. Additionally, we found no evidence of metachronous ovarian cancer in this study, which corresponds with an earlier large population-based study, which reported the incidence rate of metachronous ovarian cancer was very low, as the 10-year accumulative incidence was only 0.2% [29].

We also found that OP had no impact on oncological outcomes, including both RFS and OS. This finding is consistent with that of Lyu et al. who conducted a study based on the SEER program in patients with stage I adenocarcinoma or adenosquamous cervical cancer. They found that OP had no effect on either CSS (HR, 0.9; 95% CI, 0.50-1.61) or OS (HR, 0.77; 95% CI, 0.35-1.73) [24]. In 2016, Chen et al. also reported the same results. There was also no significant difference in disease-free survival (DFS) (*P* = 0.423) or OS (*P* = 0.330) between patients with bilateral SO and OP. Even in the subgroup analysis of patients aged less than 45 years, they found no statistical difference in either DFS (*P* = 0.478) or OS (*P* = 0.429). In addition, the patients did not develop any ovarian relapse after 16 months of follow-up [25]. Recently, surprising data was reported by a study based on the SEER database. The study was a record of patients who were 45 years of age, or younger, with T1N0M0 cervical adenocarcinoma (1988 to 2013). The study found that the OP group had a better CSS (*P* = 0.0370) and OS (*P* = 0.0025). After adjusting for covariates, the CSS benefit of ovarian conservation was marginally significant (*P* = 0.051), and the OS benefit was still significant (*P* = 0.006). This study found a benefit for OP on oncological outcomes in early-stage adenocarcinoma cervical cancer [26]. In consideration of our results, although our study did not find any differences in OS or RFS between OP and non-OP, OS seemed to be better for OP in long-term follow-up (after 15 years). Further studies with higher populations and long-term follow-up are required to confirm these effects on oncological outcomes.

There were some limitations to our study. This was a retrospective analysis, with a relatively small number of patients, and there may have been some potential confounding biases. Menopause status, for example, was assumed from the cutoff age and was not confirmed by hormonal profiles. However, all patients in our study were treated uniformly, at a single institution, via uniform surgical techniques. Further studies with longer follow-up periods or meta-analyses should be conducted with larger populations to confirm our findings. Clinicopathologic factors that are associated with ovarian metastasis need to be clarified, as this may be useful in devising some criteria for the selection of patients for ovarian preservation. The benefit of OP in terms of cardiovascular protection or reducing mortality from cardiovascular disease could also be usefully studied.

## 5. Conclusion

We found no incidence of ovarian metastasis, synchronous or metachronous ovarian cancer, or ovarian recurrence in our study, from which we conclude that ovarian preservation may be safe in patients with adenocarcinoma and adenosquamous cervical cancer stages IA2-B1. However, the impact of ovarian preservation on oncological outcomes needs to be further investigated.

## Figures and Tables

**Table 1:** Characteristic comparison between patients aged <50 years with or without ovarian preservation.

Characteristics	Total (*N* = 196)	OP (*N* = 108)	Non-OP (*N* = 88)	*P* value
Age	41 (36, 45)	38 (35, 42)	45 (41, 47)	<0.001
FIGO stage				0.033
IA2	25 (12.8)	8 (7.4)^a^	17 (19.3)	
IB1 < 2cm	71 (36.2)	44 (40.7)^b^	27 (30.7)	
IB1 ≥ 2cm	100 (51.0)	56 (51.9)^a,b^	44 (50.0)	
Histology				0.550
Adenocarcinoma	167 (85.2)	94 (87.0)	73 (83.0)	
Adenosquamous	29 (14.8)	14 (13.0)	15 (17.0)	
LVSI				0.566
No	169 (86.2)	95 (88.0)	74 (84.1)	
Yes	27 (13.8)	13 (12.0)	14 (15.9)	
Parametrial invasion				0.349
No	186 (94.9)	104 (96.3)	82 (93.2)	
Yes	10 (5.1)	4 (3.7)	6 (6.8)	
LN metastasis				0.703
No	189 (96.4)	105 (97.2)	84 (95.5)	
Yes	7 (3.6)	3 (2.8)	4 (4.5)	
Vaginal margin involvement				1.000
No	192 (98.0)	106 (98.1)	86 (96.7)	
Yes	4 (2.0)	2 (1.9)	2 (2.3)	
DSI				1.000
No	154 (78.6)	85 (78.7)	69 (78.4)	
Yes	42 (21.4)	23 (21.3)	19 (21.6)	
Type of surgery				0.254
Open	193 (98.5)	105 (97.2)	88 (100)	
Laparoscopic	3 (1.5)	3 (2.8)	0 (0)	
Adjuvant treatment				0.092
No	171 (87.2)	99 (91.7)	72 (81.8)	
RT	19 (9.7)	6 (5.6)	13 (14.8)	
CCRT	6 (3.1)	3 (2.8)	3 (3.4)	
Year of treatment				0.008
1987-1999	56 (28.6)	22 (20.4)	34 (38.6)	
2000-2017	140 (71.4)	86 (79.6)	54 (61.4)	
Postoperative HRT				<0.001^∗^
No	143 (73.0)	93 (86.1)	50 (56.8)	
Yes	27 (13.8)	6 (5.6)	21 (23.9)	
Missing	26 (13.2)	9 (8.3)	17 (19.3)	
Recurrence				0.712
No	173 (88.3)	94 (87.0)	79 (89.8)	
Yes	23 (11.7)	14 (13.0)	9 (10.2)	
Recurrent site				0.933
Locoregional	16 (69.5)	10 (71.4)	6 (66.7)	
Distance	5 (21.7)	3 (21.4)	2 (22.2)	
Combined	2 (8.7)	1 (7.1)	1 (11.1)	

Values are presented as number (%), median (IQR1-IQR3). FIGO: The International Federation of Gynecology and Obstetrics; OP: ovarian preservation; LVSI: lymph-vascular space invasion; LN: lymph node; DSI: deep stromal invasion; RT: radiotherapy; CCRT: concurrent chemoradiation; HRT: hormonal replacement therapy. ^a,b^FIGO stage not having a superscript in common within clinicopathological characteristics differ significantly (*P* value < 0.05).

**Table 2:** Univariate analysis of 5-year recurrence-free survival and 5-year overall survival in patients aged <50 years.

Characteristic	5-year RFS (95% CI)	*P* value	5-year OS (95% CI)	*P* value
FIGO stage		0.01		0.043
IA2	100.0^a^		100.0^a^	
IB1 < 2cm	93.1 (86.7-99.9)^a^		95.6 (89.7-100)^a,b^	
IB1 ≥ 2cm	85.7 (78.1-94.0)		97.3 (93.6-100)^b^	
Histology		0.254		0.513
Adenocarcinoma	91.5 (86.7-96.5)		97.3 (94.4-100.0)	
Adenosquamous	80.2 (64.4-100.0)		95.2 (86.6-100.0)	
LVSI		0.042		0.016
No	90.9 (86.0-96.0)		96.7 (93.5-100.0)	
Yes	82.2 (65.5-100.0)		100 (100-100.0)	
Parametrial invasion		<0.001		<0.001
No	90.7 (85.9-95.7)		96.8 (93.8-100.0)	
Yes	77.1 (53.5-100.0)		100 (100-100.0)	
LN metastasis		<0.001		<0.001
No	92.3 (87.9-96.8)		97.8 (95.3-100.0)	
Yes	38.1 (13.7-100.0)		80.0 (51.6-100.0)	
Vaginal margin involvement		<0.001		0.213
No	90.3 (85.5-95.3)		97.0 (94.1-100.0)	
Yes	75.0 (42.6-100.0)		100.0	
DSI		0.004		0.003
No	91.8 (87.0-96.8)		96.3 (92.9-99.9)	
Yes	81.8 (68.4-98.0)		100.0	
Type of surgery		0.940		1
Open	89.9 (85.1-94.9)		97.0 (94.2-100.0)	
Laparoscopic	100.0 (100-100.0)		100.0 (100.0-100.0)	
Adjuvant treatment		<0.001		<0.001
No	92.3 (87.8-97.1)		97.5 (94.7-100.0)	
Yes	75.2 (58.4-96.8)		94.4 (84.4-100.0)	
Postoperative HRT		0.892		0.631
No	91.2 (85.7-96.9)		97.9 (95.1-100.0)	
Yes	86.7 (73.7-100.0)		100 (100-100.0)	
Missing	86.5 (73.4-100.0)		89.2 (76.0-100.0)	
Year of treatment		0.778		0.503
1987-1999	88.0 (79.5-97.5)		95.8 (90.2-100.0)	
2000-2017	91.2 (85.7-96.9)		97.7 (94.6-100.0)	
Ovarian preservation		0.363		0.974
No	91.1 (84.4-98.3)		96.5 (91.8-100.0)	
Yes	88.9 (82.2-96.1)		97.5 (94.2-100.0)	

Values are presented as percentage (95% confidence interval). FIGO: The International Federation of Gynecology and Obstetrics; OP: ovarian preservation; LVSI: lymph-vascular space invasion; LN: lymph node; DSI: deep stromal invasion; HRT: hormonal replacement therapy. ^a,b^FIGO stage not having a superscript in common within RFS and OS differ significantly (*P* value < 0.05).

**Table 3:** Multivariate analysis of 5-year recurrence-free survival in patients aged <50 years.

Characteristics	Full model			Reduced model		
	HR	95% CI	*P* value	HR	95% CI	*P* value
FIGO stage			0.023			0.005
IA2	1			1		
IB1 < 2cm	Inf	(0-Inf)		Inf	(0-Inf)	
IB1 ≥ 2cm	Inf	(0-Inf)		Inf	(0-Inf)	
LVSI			1.000			
No	1	—				
Yes	0.99	0.22-4.48				
Parametrial invasion			0.596			
No	1	—				
Yes	1.47	(0.36-6.11)				
LN metastasis			0.018			<0.001
No	1	—		1	—	
Yes	5.15	1.39-19.10		9.77	(3.32-28.72)	
Vaginal margin involvement			0.298			
No	1	—				
Yes	2.61	0.45-15.18				
DSI			0.526			
No	1	—				
Yes	0.66	0.18-2.44				
Adjuvant treatment			0.316			
No	1	—				
RT	2.73	0.67-11.13				
CCRT	3.82	0.37-38.97				
Ovarian preservation			0.454			0.529
No	1	—		1	—	
Yes	0.72	0.30-1.73		0.76	0.33-1.78	

FIGO: The International Federation of Gynecology and Obstetrics; LVSI: lymph-vascular space invasion; LN: lymph node; DSI: deep stromal invasion; RT: radiotherapy; CCRT: concurrent chemoradiation.

**Table 4:** Multivariate analysis of 5-year overall survival in patients aged <50 years.

Characteristics	Full model			Reduced model		
	HR	95% CI	*P* value	HR	95% CI	*P* value
FIGO stage			0.037			0.014
IA2	1			1		
IB1 < 2cm	Inf	(0-Inf)		Inf	(0-Inf)	
IB1 ≥ 2cm	Inf	(0-Inf)		Inf	(0-Inf)	
LVSI			0.802			
No	1	—				
Yes	1.22	0.26-5.73				
Parametrial invasion			0.835			
No	1	—				
Yes	1.18	0.25-5.67				
LN metastasis			0.022			<0.001
No	1	—		1	—	
Yes	8.21	1.30-51.77		5.2	(1.30-21.40)	
DSI			0.481			
No	1	—				
Yes	0.56	0.11-2.97				
Adjuvant treatment			0.127			
No	1	—				
RT	3.24	0.52-20.07				
CCRT	12.4	1.09-140.34				
Ovarian preservation			0.703			0.966
No	1	—		1	—	
Yes	0.82	0.29-2.32		0.98	0.36-2.65	

FIGO: The International Federation of Gynecology and Obstetrics; LVSI: lymph-vascular space invasion; LN: lymph node; DSI: deep stromal invasion; RT: radiotherapy; CCRT: concurrent chemoradiation.

## Data Availability

The data used to support the findings of this study are available from the corresponding author upon request.
